# Comparative Anatomy of the Coracobrachialis Muscle: Insights into Human Typical and Variant Morphology

**DOI:** 10.3390/biology14091113

**Published:** 2025-08-22

**Authors:** George Triantafyllou, Alexandros Samolis, Ingrid C. Landfald, Łukasz Olewnik, Judney C. Cavalcante, Maria Piagkou

**Affiliations:** 1 Department of Anatomy, School of Medicine, Faculty of Health Sciences, National and Kapodistrian University of Athens, 11527 Athens, Greece; georgerose406@gmail.com (G.T.); alexsamolis@me.com (A.S.); 2 ”VARIANTIS” Research Laboratory, Department of Clinical Anatomy, Masovian Academy in Płock, 09400 Płock, Poland; ingridceciliee@gmail.com (I.C.L.); lukaszolewnik@gmail.com (Ł.O.); judney.cavalcante@ufrn.br (J.C.C.); 3 Department of Clinical Anatomy, Masovian Academy in Płock, 09400 Płock, Poland; 4 Group of Study and Research in Human Anatomy (NEPAH), Laboratory of Anatomy, Department of Morphology, Biosciences Center, Federal University of Rio Grande do Norte, Natal 59078-970, Brazil

**Keywords:** coracobrachialis muscle, morphological variation, evolution, comparative anatomy, developmental anatomy

## Abstract

The coracobrachialis muscle (CB) is a phylogenetically conserved component of the anterior compartment of the upper limb, offering critical insights into evolutionary anatomy, developmental biology, and human morphological variation. This mini review synthesizes findings from comparative anatomical studies, embryological data, cadaveric dissections, and clinical reports to explore the CB’s morphology, ontogeny, and medical relevance. Among tetrapods, the CB consistently originates from the coracoid process and inserts into the humerus, with interspecies morphological adaptations reflecting specific locomotor functions such as climbing, grasping, or digging. In humans, embryological studies confirm a common developmental origin with the biceps brachii, accounting for the frequent occurrence of variant configurations, including multiple heads and accessory slips such as the coracobrachialis longus (CBL) and brevis (CBB). These variants may contribute to clinical conditions such as musculocutaneous nerve entrapment+ or subcoracoid impingement. They may also be employed in reconstructive surgical procedures. A deeper understanding of CB morphology within an evolutionary framework improves anatomical interpretation, enhances clinical safety, and reduces diagnostic ambiguity. This review highlights the significance of integrating evolutionary and developmental perspectives in the study of human muscle variation.

## Simple Summary

The coracobrachialis muscle (CB) is a small upper arm muscle involved in shoulder movement and stabilization. This paper explores its anatomical variations, development, and evolutionary history across vertebrates. In humans, the CB often shows multiple heads and variable nerve pathways, commonly involving the musculocutaneous nerve. These variants have clinical significance, especially in surgery and diagnostics, due to risks of nerve entrapment or misidentification. Embryological studies suggest a shared origin with the biceps brachii. Comparative anatomy shows that CB variants reflect evolutionary remnants from ancestral species, emphasizing the muscle’s importance in both evolutionary biology and modern clinical practice.

## 1. Introduction

Human anatomical variation encompasses a broad morphological spectrum across muscles, bones, and neurovascular structures. These variations hold substantial clinical relevance, particularly during surgical procedures, where unexpected anatomical configurations may complicate interventions or lead to iatrogenic injuries. Therefore, comprehensive knowledge of both common and rare anatomical variants is essential for safe and effective clinical practice [1].

Several muscular variations can be justified through comparative anatomy, which examines homologous structures across species to infer evolutionary relationships [2] and developmental anatomy, which explores how these structures emerge and differentiate during embryogenesis [3]. One such example is the coracobrachialis muscle (CB), a slender muscle of the anterior arm compartment that is frequently subject to morphological variations.

In the anterior arm compartment, three primary muscles are identified: the biceps brachii (BB), the brachialis (B), and the CB. All of these are innervated by branches of the musculocutaneous nerve (MCN). The CB typically originates from the coracoid process of the scapula, sharing this proximal attachment with the short head of the BB. Its distal insertion is located along the medial surface of the humeral shaft [4]. However, numerous variations in origin, insertion, number of heads, and innervation patterns have been documented across populations, as well as within individuals [1].

Developmentally, these variants can arise due to incomplete muscle differentiation, fusion anomalies, or the persistence of embryonic muscle primordia [5]. Such developmental mechanisms not only explain the morphological diversity of the CB but also its potential implications for neurovascular entrapment syndromes and surgical risk [6].

This comprehensive review presents the comparative anatomy, embryology, and phylogenetic significance of the CB, linking non-human and human muscular patterns. These details can significantly improve our understanding of the morphogenesis and evolutionary trajectory of this muscle, while emphasizing the clinical significance of its variants in orthopedic, reconstructive, and neurovascular surgical contexts.

## 2. Coracobrachialis Muscle Anatomy Across Vertebrates

The CB is a consistent and evolutionarily conserved part of tetrapod forelimb muscles. It typically originates from the coracoid process and inserts on the medial humeral shaft. In tetrapods, the coracobrachialis muscle plays a crucial role in adducting and stabilizing the shoulder joint. Its precise anatomical configuration and functional contributions vary across species, reflecting diverse locomotive adaptations and ecological niches.

This section combines comparative anatomical data from amphibians, reptiles, birds, monotremes, therians, and primates, with a focus on innervation patterns, developmental origins, and functional changes. Studying the CB across different groups not only shows its evolutionary history, but also helps in interpreting human morphological variations as either inherited or reactivated ancestral traits.

### 2.1. Non-Amniote Tetrapods

Among non-amniote tetrapods, the CB is already a morphologically distinct muscle that often appears divided into multiple heads. In urodeles such as *Ambystoma*, the CB typically separates into a coracobrachialis superficialis (CBS) and a coracobrachialis profundus (CBP). This segmentation likely reflects early muscular differentiation from the ventral limb bud musculature [7].

In the anuran *Rhinella*, a similar arrangement occurs, although the two components may fuse or appear less distinct [7]. These patterns are consistent with general evolutionary trends in forelimb muscle homology among early tetrapods.

### 2.2. Reptiles and Birds

**Figure 1:**
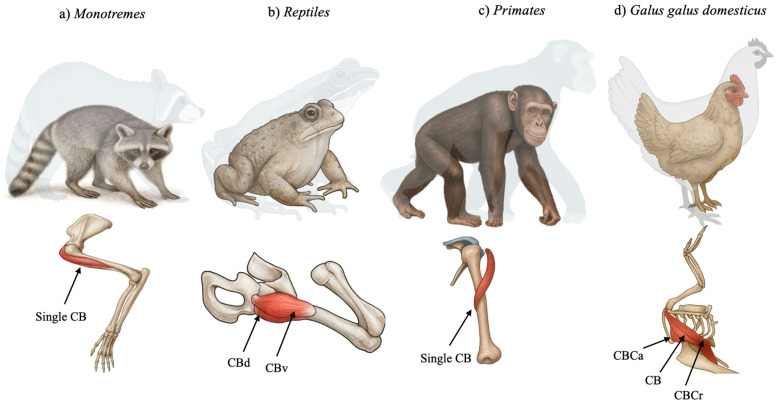
Schematic representation of the coracobrachialis muscle (CB) among different species. CBd—coracobrachialis dorsalis, CBv—coracobrachialis ventralis, CBCa—coracobrachialis caudalis, and CBCr—coracobrachialis cranialis.

In many groups of reptiles, such as turtles and lizards, CB is described as a duplicated muscle represented by the CBB and the CBL. In crocodiles, only the CBB has been described, divided into CBB ventralis and CBB dorsalis (Figure 1), with functions including flexion, adduction, and the stabilization of the shoulder joint. In aquatic Cheloniidae, CB can promote the depression and retraction of the anterior limb, important for swimming [8].

In birds, the CB has undergone significant remodeling. Koizumi (2024) [9] demonstrated that in *Gallus gallus domesticus* (domestic chicken), the so-called “coracobrachialis group” includes three distinct muscles: CB cranialis (CBCr), CB caudalis (CBCa), and a third unnamed muscle that aligns most closely with the classical CB (Figure 1). CBCr and CBCa are innervated by the axillary and pectoral nerves, respectively, suggesting homology not with the ancestral CB, but with the deltoid and pectoralis muscles [9]. Only the third muscle, which originates from the acrocoracoid and inserts onto the dorsal tubercle of the humerus, matches the traditional CB structure [9]. This revised classification, based on detailed neuroanatomical mapping, reflects the adaptive transformations associated with flight and challenges long-held assumptions regarding avian muscle homology [9].

### 2.3. Monotremes

In monotremes, the CB exhibits a morphologically conservative anatomy (Figure 1). Regnault et al. [10] investigated the shoulder musculature of the short-beaked echidna (*Tachyglossus aculeatus*) through contrast-enhanced CT and gross dissection. Their analysis confirmed a classical CB configuration, originating from the fused coracoid region and inserting into the medial humerus. The muscle exhibited long fascicles and a moderate physiological cross-sectional area, suggesting a design optimized for broad-range motion rather than high-force generation. This morphology supports a sprawling gait and the burrowing behavior typical of echidnas. Its lack of architectural specialization further supports its classification as a primitive mammalian trait [10].

### 2.4. Therians and Non-Primate Mammals

Among therian mammals, the CB usually appears as a single-headed muscle, although multiple slips or accessory heads can occur in some taxa, such as rodents and primates. In carnivorans, García et al. [11,12] clarified the distinction between the true CB and a second muscle historically referred to as the CBB or articularis humeri. Their dissections and innervation tracing in *Procyon cancrivorus* and *Nasua nasua* revealed the following:

- The true CB is located medially, originates from the coracoid process, and is innervated by the MCN;
- The axillary nerve innervates the second, more posterior muscle and likely derives from the subscapularis group, rather than being a CB variant.

These results highlight the importance of nerve supply in establishing muscular homology and underscore the need for precise anatomical nomenclature [11,12].

### 2.5. Primates

In primates, the CB shows both evolutionary conservatism and functional specialization (Figure 1). In the capuchin monkey (*Sapajus apella*), Monroy-Cendales et al. [13] reported a well-developed CB that aids in shoulder stabilization and adduction during arboreal locomotion. The muscle closely aligns with the mammalian archetype, originating from the coracoid and inserting midway along the humerus.

In great apes, Sonntag [14] described a robust and hypertrophied CB in chimpanzees, emphasizing its role in forelimb strength, brachiation, and climbing.

A neuroanatomical study by Koizumi and Sakai [15] found that the CB in apes may receive input not only from the MCN, but also from independent rami arising from the upper and middle trunks of the brachial plexus. This complex innervation pattern supports the hypothesis that the CB in apes—and by extension, in humans—serves a transitional role between arm flexors and shoulder stabilizers, reflecting its origin from a multifunctional ventral muscle mass [15].

### 2.6. Developmental Links to Human Anatomy

From a developmental perspective, the CB develops from the ventral pre-muscular mass of the embryonic limb bud. Diogo et al. [16,17,18,19] showed that it shares a common precursor field with the BB, which explains the frequent anatomical association and shared innervation with the MCN. In a study on limb regeneration in axolotls, Diogo et al. [18] observed that CB-like structures can emerge anomalously, indicating that the CB’s developmental program is robust, but also susceptible to reactivation under regenerative conditions. Molnar and Diogo [20] further emphasized the CB’s evolutionary modularity, noting its high conservation, resistance to loss, and retention of core anatomical traits—namely its medial location and MCN innervation—even in the face of significant limb evolution across vertebrates. Its persistence underscores its mechanical significance in stabilizing the shoulder joint.

## 3. Coracobrachialis Muscle Anatomy Among Humans

The CB is a key component of the anterior compartment of the upper limb. It plays a crucial role in shoulder flexion, humeral adduction, and joint stabilization, particularly during arm elevation and resisted motion. Structurally and functionally, it is one of the most consistent yet variably expressed muscles in human anatomy.

### 3.1. Embryological Development of the Human Coracobrachialis Muscle

The embryological origin of the CB reflects both its deep phylogenetic lineage and the intricate developmental processes of the upper limb musculature in humans. As observed across tetrapods, the CB arises from the ventral muscle mass of the embryonic limb bud, in close developmental association with the BB and B [5]. According to Bardeen’s classic embryological studies, the anterior arm compartment muscles—including the CB, BB, and B—originate from a common mesenchymal condensation. This primordium first appears in embryos of approximately 11 mm in length, with clear differentiation into distinct muscle bellies (such as the CB and BB) beginning around 14–16 mm [5]. Notably, the proximal segments of the muscle masses differentiate earlier than the distal regions, accounting for the tight developmental and anatomical link between the CB and the short head of the BB [5].

A pivotal modern study by Yamamoto et al. [21] analyzed serial histological sections of ten human embryos (crown–rump length: 26–32 mm), corresponding to approximately seven weeks of gestation. The findings revealed that the early CB is not yet morphologically distinct from the short head of the BB. Instead, both structures emerge from a shared muscular anlage, with the CB developing as a medial extension or differentiated subdivision of the same muscle mass [21]. This embryonic continuity contrasts with the distinct, separate appearance of the CB and BB seen in adult anatomy [4].

One of the most striking observations in the embryonic series was the trajectory of the MCN. In all examined specimens, the MCN passed between the undifferentiated muscle mass destined to become the CB and BB short head [21]. This intermuscular pathway precedes the later separation of the muscle bellies and explains the adult configuration in which the MCN pierces the CB. This observation also suggests that the nerve–muscle relationship is dynamic during development, shifting in tandem with the progressive compartmentalization of the muscular mass [21].

In embryos where the BB heads were more clearly segregated, a distinct muscle mass corresponding to the CB could be identified on the medial or anterior side of the MCN. This structure remained partially continuous with the short head of the BB, reinforcing the hypothesis that both muscles originate from a common precursor and separate secondarily [21].

An additional noteworthy anatomical variant was observed in one specimen: a thick nerve branch emerged from the MCN after its passage between the BB and CB and subsequently joined the median nerve (MN). This finding suggests that transient or accessory neural communications may exist during early brachial plexus development and could persist as rare variants in adults [21].

These embryological findings partially corroborate earlier anatomical hypotheses by Koizumi [15], who proposed that the CB in apes and humans consists of two bellies—a superficial head, innervated by direct branches from the lateral cord, and a deep head, supplied by the MCN. In this model, the MCN runs between the two layers, rather than penetrating a unified muscle. However, the data from Yamamoto et al. [21] suggest that such a bilaminar arrangement is not evident at the seven-week embryonic stage. Instead, a single, unified muscle mass initially exists, which later undergoes internal reorganization to adopt the layered or bifid configurations seen in adult and variant anatomy.

### 3.2. Evidence of Human Anatomy in Original Studies

Extensive anatomical studies have highlighted that the CB exhibits a significant degree of morphological variability. The very first two studies presented the two-headed CB as the most common configuration of the muscle, with El-Naggar [22] reporting it in all specimens (36 upper limbs) and Ilayperuma et al. [23] recording it in 83.3% of their sample (312 upper limbs).

Several years later, Szewczyk et al. [24] proposed a classification system that recognizes Type I (single head), Type II (double head with two subtypes: one originating from the coracoid process and one from the biceps tendon, or both from the coracoid process), and Type III (three heads, with two from the coracoid process and one from the biceps tendon). They found Type I in 49.5%, Type II in 42.6%, and Type III in 7.9% of their 101 upper limbs [24].

Most notably, the muscle may present one to four distinct heads of origin. In the comprehensive dissection series by Piagkou et al. [25], a two-headed CB—comprising superficial and deep heads—was the most common form, found in 62.96% of cadaveric arms. Three-headed and four-headed variants were also identified in 22.2% and 3.7% of cases, respectively, while a one-headed form occurred in 11.1%. Similar patterns were confirmed in a fetal cadaveric study by Triantafyllou et al. [26], where the two-headed form appeared in 60.7%, while single-, three-, and four-headed forms represented 18.6%, 10%, and 10.7%, respectively. Rarer cases were also described with five-headed [27] and six-headed CB [28]. For all the cases with CB variations, the accessory heads originated from the coracoid process and/or the short head of the biceps brachii, similar to the typical origin of the CB [25,26,27,28]. Different points of muscle origin were not reported to the authors’ knowledge.

The MCN’s path relative to the CB varies similarly, and often depends on the muscle’s arrangement (Table 1):

- In two-headed and multi-headed CB, the MCN typically pierces the muscle belly;
- In single-headed forms, the MCN usually follows a medial course, bypassing the muscle without penetration.

This correlation was consistently confirmed by Piagkou et al. [25] and Triantafyllou et al. [26]. Such variations are essential for clinicians, especially in brachial plexus surgeries, nerve decompression, and regional anesthesia, where precise nerve localization is critical.

**Figure 2:**
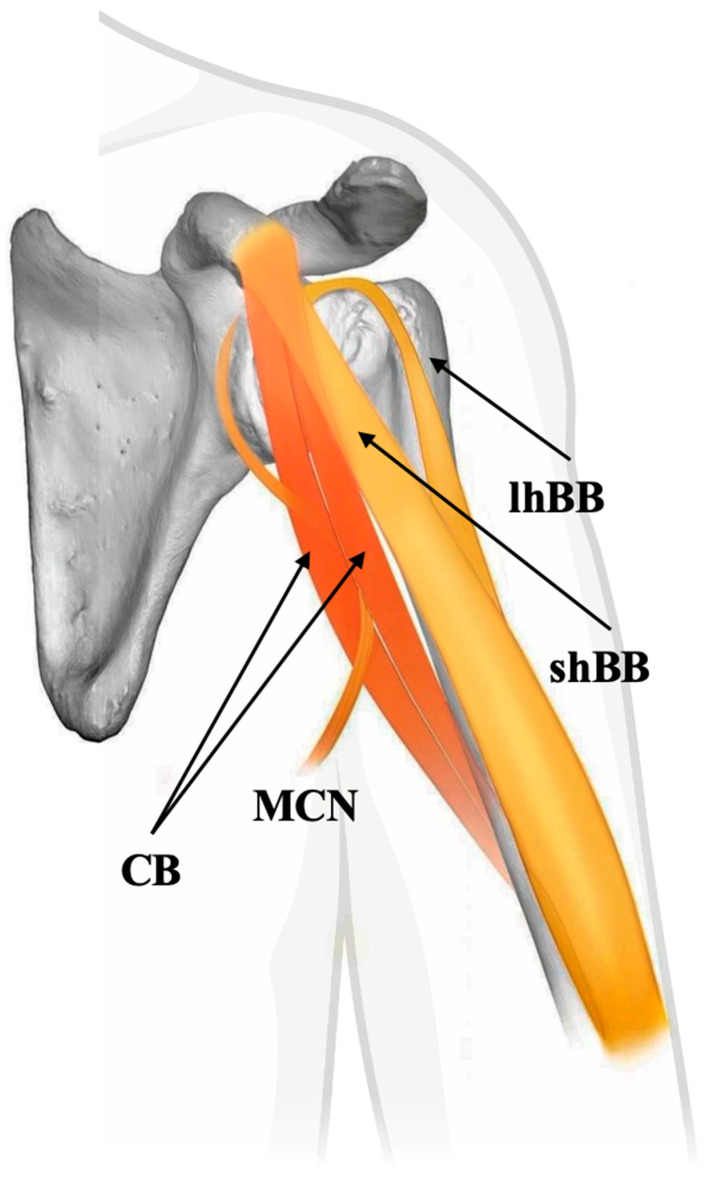
Schematic representation of the two-headed coracobrachialis muscle (CB) with the musculocutaneous nerve passing between the heads, indicative of the typical human anatomy. shBB—short head of biceps brachii muscle, lhBB—long head of biceps brachii muscle, and MCN—musculocutaneous nerve.

Recent cadaveric and embryological studies confirm that the “classical” one-headed CB described in anatomical texts is not the most common configuration. Instead, the two-headed CB, often pierced by the MCN, appears to be the default anatomical variant in both adult and fetal populations [29]. This paradigm shift highlights the importance of recognizing variability as typical rather than exceptional in muscle anatomy. These findings also support the developmental and evolutionary continuity of the CB with its more complex ancestral forms, usually formed by two muscles, as seen in other tetrapods (Figure 2).

### 3.3. Coracobrachialis Muscle: Possible Evolutionary Remnants in Human Anatomy

**Figure 3:**
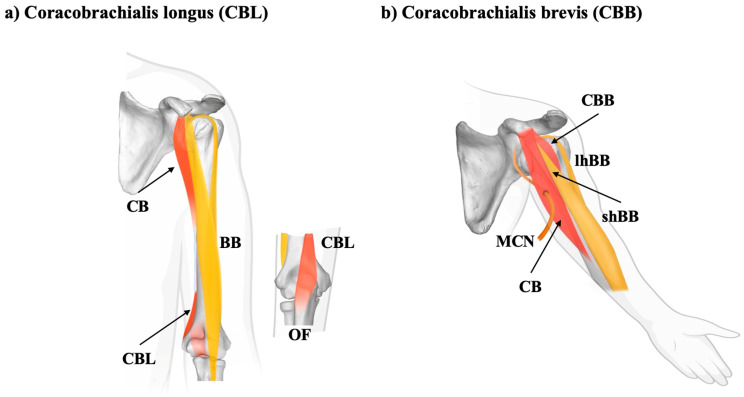
Schematic representation of the coracobrachialis longus type II (insertion into the olecranon) and brevis (CBL and CBB) variations. MCN—musculocutaneous nerve, BB—biceps brachii, shBB—short head of biceps brachii muscle, lhBB—long head of biceps brachii muscle, and OF—olecranon fossa.

The CB in humans displays a remarkable range of morphological variants, many of which are increasingly recognized as evolutionary remnants of ancestral musculoskeletal configurations. These variants, including the coracobrachialis longus (CBL), coracobrachialis brevis or superior (CBB or CBS) (Figure 3), and other lesser-known accessory slips, provide essential insights into the phylogenetic development of the upper limb and hold potential clinical relevance (Table 2). The CBL is one of the most commonly reported accessory muscles originating from the CB complex. Zielińska et al. [30], in a dissection study of 100 human upper limbs, found the CBL in 11% of cases and proposed a two-type morphological classification.:

- Type I: insertion in the medial epicondyle of the humerus;
- Type II: insertion into the olecranon.

These variants extend more distally than the typical CB, often crossing the elbow joint, suggesting that they represent a persistent homolog of more expansive brachial musculature observed in quadrupedal mammals, where the CB contributes to forelimb stabilization, weight bearing, and locomotor efficiency [30]. The unilateral presence, radial innervation, and accessory functional potential of such muscles were further demonstrated by Maslanka et al. [31], who reported a CBL innervated by the radial nerve—a rare configuration with essential implications for surgical and radiological interpretation.

Another noteworthy variation is the CBB or CBS. These are proximal accessory bellies, often inserted at or near the surgical neck of the humerus. Sugalski et al. [32] described a bilateral CBB, located deep in the CB and the short head of the BB, arising from the coracoid process. In a similar vein, Olewnik et al. [33] identified a rare CBS variant originating above the common CB-BB short head origin and passing near the lateral root of the MN before fusing distally with the CB. These topographic relationships underscore the neurovascular risks posed by such variants during surgery or trauma.

The phylogenetic relevance of these accessory structures has been emphasized by referencing early mammalian dissections by Wood [34], where multi-headed or extended CB muscles were documented in echidnas, squirrels, armadillos, and other mammals. In these species, the CB complex frequently contributed to grasping, climbing, or burrowing, and retained multiple heads with deep insertions in the ulna or joint capsule. Further complexity was introduced in the recent report by Zielińska et al. [35], who described a tripartite CB variant with two additional structures:

- A tendinous slip arising from the surgical neck of the humerus;
- A muscular band inserting in the capsule of the glenohumeral joint.

These components merged with the third head of the CB distally, potentially functioning as dynamic stabilizers of the shoulder joint, which may reflect ancestral traits related to primate forelimb control [30,33,35].

## 4. Clinical Anatomy of the Human Coracobrachialis Muscle

Beyond its traditional role in shoulder flexion and adduction, the CB has significant clinical implications, particularly due to its morphological variants. Accessory CB structures, such as the CBL and CBB or CBS, are linked to various clinical syndromes, including entrapment neuropathies and subcoracoid impingement, and have shown promise in reconstructive surgery.

### 4.1. Subcoracoid Impingement and Anterior Shoulder Pain

One of the most notable clinical signs of CB variants is subcoracoid impingement syndrome. This condition presents with anterior shoulder pain, especially during flexion, adduction, and internal rotation. Variants of the CB may occupy the coracohumeral space, resulting in the compression of nearby soft tissue structures.

- Mestdagh et al. [36] described a case involving an alpinist whose shoulder pain resulted from an accessory CB slip crossing anterior to the subscapularis. The surgical removal of the slip led to the complete relief of symptoms.
- Bauones et al. [6] identified accessory CB-related subcoracoid impingement using dynamic ultrasound, highlighting the importance of real-time imaging for diagnosing these mobile variants.

Recent imaging studies support these findings. In a prospective cohort of 664 patients, Bauones et al. [6] documented a 1.04% prevalence of accessory CB muscles, which were more common in females. The accessory slip was consistently located above the subscapularis and deep into the deltoid, appearing as a hypoechoic structure with echogenic septa on ultrasound (US), and variably visible on MRI. Ultrasound proved more sensitive due to its real-time dynamic ability and higher soft tissue contrast for superficial structures [6].

### 4.2. Nerve and Vascular Entrapment Syndromes

CB variants can be directly implicated in neurovascular entrapment, especially involving the MCN and, in some cases, the brachial artery:

- El-Naggar [22] documented a CB variant forming a muscular tunnel that is traversed by both the MCN and brachial artery, potentially increasing the risk of entrapment neuropathy.
- Piagkou et al. [25] highlighted the dangers of multi-headed CB variants, especially when they are near the MCN during regional anesthesia or surgical procedures.

### 4.3. Role in Reconstruction and Microsurgery

In reconstructive surgery, the CB’s favorable vascular anatomy and proximity to neurovascular bundles make it a valuable flap donor:

- Hobar et al. [37] reported the successful use of a CB muscle flap to cover exposed axillary vessels after radical dissection. The flap, based on brachial artery perforators, provided strong vascularity and minimal donor site morbidity.
- Taylor et al. [38] recommended the CB for facial reanimation surgeries, owing to its ideal length, flexibility, and reliable neurovascular pedicle, especially in cases needing muscle transfer to restore facial expression.

### 4.4. Radiological and Surgical Implications

Awareness of CB variants is crucial to prevent misdiagnosis or intraoperative complications. Accessory CB slips may resemble soft tissue tumors, vascular malformations, or nerve sheath tumors visible on MRI, as well as pseudomasses seen on ultrasound, especially when hypertrophic or fibrotic [30,35]. Zielińska et al. [30] warned that a CBL inserting into the olecranon could be mistaken for accessory triceps slips or myofascial abnormalities, especially in posterior elbow imaging. In surgical dissection, accessory CB structures can obscure the MCN, confuse surgical planes, or mislead flap design, particularly in axillary and brachial approaches. Surgeons performing nerve decompression, vascular access, or orthopedic repair around the coracoid process must remain vigilant for such variants [30].

## 5. Conclusions

The CB offers a compelling example of how comparative anatomy enhances our understanding of human muscular morphology and variation. Present in nearly all tetrapods, the CB exhibits a conserved evolutionary framework, yet displays lineage-specific modifications in reptiles, birds, monotremes, and primates that reflect shifts in forelimb function and locomotor demands. In humans, while the CB maintains its typical origin from the coracoid process and insertion in the humerus, it is notably prone to morphological variability, including accessory heads, variant insertions, and distinct neuromuscular relationships. These human variants—such as the CBL and CBB/CBS—closely parallel muscular patterns observed in other mammals, suggesting the retention of ancestral morphogenetic pathways. Their frequent co-development with the BB further supports a shared embryological origin, emphasizing the value of the CB as a model system in evolutionary developmental anatomy (evo-devo). From a clinical perspective, CB variants are not rare curiosities, but anatomically and surgically significant structures implicated in neurovascular entrapment, subcoracoid impingement, and flap design in reconstructive surgery.

## Figures and Tables

**Table 1:** Comparative overview of coracobrachialis (CB) innervation patterns across major vertebrate groups. While the musculocutaneous nerve (MCN) is the predominant nerve in mammals, variation and accessory innervation are common in primates and certain carnivores. This variability highlights both the evolutionary plasticity and clinical relevance of CB neuroanatomy.

Species/Clade	CB Innervation	Innervation Implication	Reference
Urodeles (*Ambystoma*)	Limb plexus (primitive)	Conserved early neuromuscular pattern	Abdala and Diogo [7]
Birds (*Gallus gallus*)	Axillary/pectoral/unknown	Suggests homology with deltoid and pectoralis; CB is often reduced	Koizumi [9]
Monotremes (*Echidna*)	MCN	Reflects a primitive mammalian brachial innervation pattern	Regnault et al. [10]
Carnivores (*Procyon*, *Nasua*)	MCN (CB), axillary (accessory muscle)	Highlights accessory muscle innervation and naming inconsistencies	García et al. [11,12]
Great Apes/Humans	MCN (and accessory rami in apes)	Dual or variable innervation noted; clinically significant	Koizumi and Sakai [15]

**Table 2:** Documented human coracobrachialis muscle (CB) variants, including the coracobrachialis longus (CBL) and brevis/superior (CBS), highlighting their anatomical features, variable innervation, and implications for clinical diagnosis, surgical procedures, and evolutionary interpretation. MCN—musculocutaneous nerve.

Muscle Variant	Origin	Insertion	Innervation	Clinical/Evolutionary Notes	References
Coracobrachialis longus (CBL)	Coracoid process	Medial epicondyle/olecranon	MCN/radial (rare)	Evolutionary remnants resembling distal brachial musculature may cause impingement or mimic soft-tissue lesions	Zielińska et al. [30] Maslanka et al. [31]
Coracobrachialis brevis (CBB)	Coracoid process	Surgical neck of the humerus	MCN or variant branch	Proximal slip may represent an atavistic remnant of the deltoid or early CB configuration	Sugalski et al. [32] Olewnik et al. [33]

## Data Availability

All the data are available upon reasonable request to the corresponding author.
